# Correlation of the gut microbiome and immune-related adverse events in gastrointestinal cancer patients treated with immune checkpoint inhibitors

**DOI:** 10.3389/fcimb.2023.1099063

**Published:** 2023-03-03

**Authors:** Yifan Zhang, Siyuan Cheng, Hua Zou, Zihan Han, Tong Xie, Bohan Zhang, Die Dai, Xiaochen Yin, Yong Liang, Yan Kou, Yan Tan, Lin Shen, Zhi Peng

**Affiliations:** ^1^ Department of Gastrointestinal Oncology, Key Laboratory of Carcinogenesis and Translational Research (Ministry of Education/Beijing), Peking University Cancer Hospital and Institute, Beijing, China; ^2^ Department of Medical Oncology and Radiation Sickness, Peking University Third Hospital, Beijing, China; ^3^ Xbiome, Shenzhen, China; ^4^ Department of Colorectal Surgery, China-Japan Friendship Hospital, Beijing, China

**Keywords:** immune-related adverse event, gut microbiome, gastrointestinal cancer, metagenome sequencing, probiotic

## Abstract

**Introduction:**

The wide application of immune checkpoint inhibitors has significantly improved the survival expectation of cancer patients. While immunotherapy brings benefits to patients, it also results in a series of immune-related adverse events (irAEs). Increasing evidence suggests that the gut microbiome is critical for immunotherapy response and the development of irAEs.

**Methods:**

In this prospective study, we recruited 95 patients with advanced/unresectable gastrointestinal cancers treated with immunotherapy and report a comprehensive analysis of the association of the gut microbiome with irAEs. Metagenome sequencing was used to analyze the differences in bacterial composition and metabolic pathways of baseline fecal samples.

**Results:**

In summary, we identified bacterial species and metabolic pathways that might be associated with the occurrence of irAEs in gastric, esophageal, and colon cancers. Ruminococcus callidus and Bacteroides xylanisolvens were enriched in patients without severe irAEs. Several microbial metabolic pathways involved in the urea cycle, including citrulline and arginine biosynthesis, were associated with irAEs. We also found that irAEs in different cancer types and toxicity in specific organs and the endocrine system were associated with different gut microbiota profiles. These findings provide the basis for future mechanistic exploration.

## Introduction

The wide usage of immunotherapy has drastically changed the cancer treatment landscape in recent years. Immunotherapy aims to boost host immune system to fight cancer by blocking immune checkpoints, which significantly improves the long-term survival and life quality of cancer patients (Liu et al., 2021). While immunotherapy can activate the immune system, it may also produce unique therapeutic toxicities known as immune-related adverse events (irAEs). IrAEs are characterized by high incidence, unknown mechanisms, and are difficult to predict. Studies have shown that the overall incidence of irAEs ranges from 54% to 76% (Xu et al., 2018). Although most irAEs tend to be mildly toxic and self-limited, severe irAEs still occur in 5-30% patients, limiting their therapeutic benefit. Therefore, understanding the mechanism of irAEs, developing effective predictive markers, and formulating individualized strategies to prevent and manage irAEs have become urgent issues for physicians.

On the biomarker discovery aspect, recent studies have proposed several potential biomarkers of irAEs, including body composition parameters (Daly et al., 2017), circulating IL-17 (Tarhini et al., 2015), IL-10 (Sun et al., 2008), CD163 (Fujimura et al., 2018), and eosinophil counts (Nakamura et al., 2019; Liu et al., 2021). In addition, a growing body of preclinical and clinical evidence suggests that the gut microbiome is critical to immunotherapy response and may also influence the onset and development of irAEs (Vétizou et al., 2015; Bhatt et al., 2017; Batten et al., 2019; Andrews et al., 2021; Liu et al., 2021; Bredin and Naidoo, 2022; Tan et al., 2022).. Previous studies in melanoma patients have shown that gut bacterial diversity (Batten et al., 2019), specific microbial quantities (such as Bacteroidetes (Dubin et al., 2016; Chaput et al., 2017), *Bacteroides vulgatus* and *Bacteroides dorei* (Usyk et al., 2021)) and related microbial-derived products (such as systemic and intestinal lipopolysaccharide (McCulloch et al., 2022)) are closely associated with the occurrence and/or severity of irAE. In a study of combined CTLA-4 and PD-1 blockade treated cohort, Miles C et al. showed irAEs could be distinguished by the higher abundance of *Bacteroides intestinalis* and *Intestinibacter bartlettii* and further demonstrated in a murine model that *Bacteroides intestinalis* was closely associated with host intestinal IL-1β and immunotherapy-related enterotoxicity (Andrews et al., 2021). One study on non-small cell lung cancer patients also identified microbial biomarkers associated with clinical efficacy and irAE severity, including *Agathobacter, Lactobacillus* and *Raoultella* etc (Hakozaki et al., 2020).

The above-mentioned studies mainly focus on melanoma and lung cancer patients, for whom immunotherapy shows an overall encouraging efficacy. They provide a solid base on the relationship between the gut microbiome and irAEs. For gastrointestinal (GI) cancers, the relationship between the gut microbiota and tumor immune microenvironment is physically closer and metabolically more complex. Several studies have shown that gut microbiota composition is related to the occurrence and development of GI cancers such as colon cancer (Feng et al., 2015; Hashemi Goradel et al., 2019; Akkız, 2021). In 2020, our group further showed a close association between the gut microbiome and immunotherapy efficacy (Peng et al., 2020). Considering the heterogeneity of microbial biomarkers across cancer types and the high incidence and mortality rates of GI cancer in Asia, we believe it is essential to explore and understand how the gut microbiome is involved in the process of irAEs in GI cancer patients. To achieve this goal, we recruited a cohort of GI cancer patients receiving immunotherapy. By analyzing their gut microbiome before treatment using metagenomics, we identified a number of microbes that are closely associated with irAEs and could be potential predictive biomarkers and/or therapeutic targets.

## Materials and methods

### Patient recruitment and clinical evaluation

**Figure 1:**
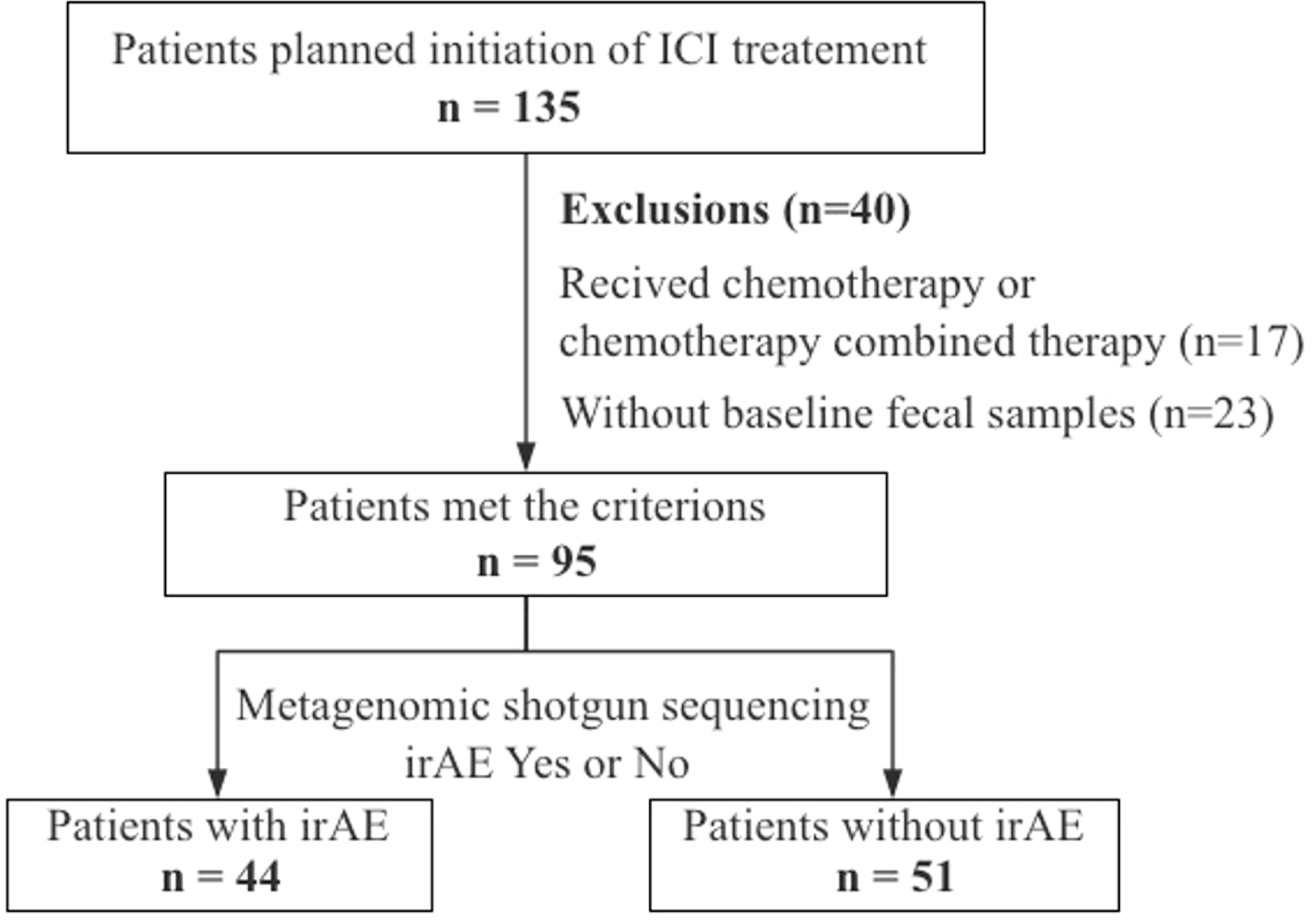
Study workflow.

A total of 135 patients with advanced/unresectable gastrointestinal cancers (esophageal cancer, gastric cancer, and colon cancer) who were hospitalized and scheduled for immunotherapy in Beijing Cancer Hospital from March 2018 to July 2021 were included in this study. The study was conducted under Institutional Review Board (IRB)–approved protocols (2018KT66) and complied with the declaration of Helsinki. All patients were fully informed about the research content and signed the consent. Final 95 patients were included for analysis because 17 patients were treated with combined immunotherapy and 23 patients failed to provide baseline fecal samples (**Figure 1**). Baseline fecal samples were defined as fecal samples collected before the start of immunotherapy or within 3 weeks of the first infusion of immunotherapy. All patients received the following two treatment regimens without antibiotic use during the treatment until disease progression or intolerable toxicity: 1) PD-1/PD-L1 inhibitor, repeated every 2 or 3 weeks; 2) combined PD-1/PD-L1 inhibitor and CTLA-4 inhibitor immunotherapy, repeated every 3 or 6 weeks.

Treatment responses were evaluated according to the Response Evaluation Criteria in Solid Tumors (RECIST).1.1 standard. Cases of irAEs were graded according to the Common Terminology Criteria for Adverse Events 4.03 (CTCAE v4.03), and the irAE scores were reviewed by at least two oncologists. The grade of irAEs was scored from 1 to 5 and was divided into mild (grade 1–2) and severe (grade 3–5) or low (grade 1) and high (grade2-5). IrAEs were limited to adverse events that were definitely or most likely linked to the treatment of immunotherapy. Patients with no irAEs were confirmed at the last clinical visit. Patients’ demographic and clinical information were collected and summarized in **Table 1**, including age, gender, diagnosis, microsatellite status, allergy history, and combined medication history etc.

**Table 1:** Demographic and clinical information of enrolled patients.

Clinical factor		Total (n=95)	irAE-Yes (n=44)	irAE-No (n=51)	*P* value (fisher test)
**Gender**	Male	69	33	36	0.653
	Female	26	11	15	
**Diagnosis**	Esophageal Cancer	27	15	12	0.083
	Gastric Cancer	35	11	24	
	Colorectal Cancer	33	18	15	
**Age**	< 60	54	22	32	0.221
	>= 60	41	22	19	
**BMI**	Normal	64	32	32	0.381
	Non-Normal	31	12	19	
**Allergy History**	Yes	6	4	2	0.411
	No	89	40	49	
**Tumor Stage**	Stage III	7	2	5	0.445
	Stage IV	88	42	46	
**ECOG**	<2	91	43	48	0.621
	>=2	4	1	3	
**MSI**	MSI High	42	21	21	0.542
	Non-MSI High	53	23	30	
**Prior Treatment**	>= 3	35	20	15	0.136
	<3	60	24	36	
**Treatment**	Anti-PD-1 Anti-PD-L1 Anti-PD1+anti_CTLA4	69 18 8	29 10 5	40 8 3	0.405

### Fecal DNA extraction and metagenomic shotgun sequencing

Baseline fecal samples were collected using the Wehealthgene^®^ Fecal Microlution™ Collection Kit (Catalog No. ML-001A, Wehealthgene). All fresh fecal samples were stored in sterile containers at -20°C and transported to the sequencing facility. Fecal DNA was extracted according to the protocol provided in the QIAamp PowerFecal DNA Kit (Cat. No. 12830-50, Qiagen). NEBNext Ultra DNA Library Prep Kit was used to construct individual sequencing library for each sample. The pooled library was sequenced by the Illumina NovoSeq 6000 sequencing platform according to manufacturer’s protocol on 150 bp paired-end reads (Novo Gene, China).

Metagenome sequences were first quality controlled to remove low-quality reads and contaminated host reads. Specifically, Fastp (version 0.20.0) and KneadData (version 0.6.1) was applied to remove reads either low in quality or read length with trimmomatic-options “ILLUMINACLIP:adapter:2:40:15 SLIDINGWINDOW:4:20 MINLEN:50”. The resulting reads were further filtered to remove host reads using Bowtie2 (bowtie2-options –very-sensitive –dovetail; hg19 version of the human genome) (**Supplementary Table 1**).

For bacterial taxa and functional profiling, HUMAnN2 (Franzosa et al., 2018) (version 2.2.0) was applied. Specifically, previously generated high-quality reads from each sample were classified using a marker-gene based approach through MetaPhlAn2 (Truong et al., 2015) with default parameters (version 2.2.0). The resulting species relative abundance were listed in **Supplementary Table 2**. Functional profiles were conducted by mapping reads to the pangenomes of species identified by MetaPhlAn2. The coding sequences of proteins were annotated in UniRef 90. Unmapped reads were translated and mapped to UniRef90 by DIAMOND (Buchfink et al., 2015). Reads which failed to map to the pangenomes of known species were labeled as “unclassified”. Gene families were analyzed to reconstruct metabolic pathway based on the MetaCyc databases (Caspi et al., 2018). The resulting metabolic pathway abundance were listed in **Supplementary Table 3**.

### Statistical analyses

Patients’ demographic and clinical information was compared using Fisher’s exact test to assess the association between patients’ demographic/clinical characteristics and immune-related adverse events. For gut microbial community analysis, alpha diversity (represented by Shannon index and inverse Simpson) and beta diversity (calculated by Bray-Curtis distance) were analyzed. To identify potential confounders, we applied permutational multivariate analysis of variance (PERMANOVA) (Anderson, 2014), a distance-based method that tests for association between microbiome and environmental factors of interest. To identify differential microbial taxa or metabolic pathways between comparison groups, MaAsLin2 (version 1.7.3) (Mallick et al., 2021) was used with the following parameters (min_abundance=0.0, min_prevalence=0.1, min_variance=0.0, normalization=“NONE”, transform=“LOG”, analysis_method=“LM”, correction=“BH”, standardize=FALSE). Microbial features were considered as significant when FDR corrected P value <0.3. Otherwise, P value less than 0.05 was considered statistically significant. The Wilcoxon rank sum test was applied for specific microbial feature comparison. All statistical analyses and plotting were performed in R (version: 3.6.3).

## Results

### Patient characteristics

This study included 27 patients with esophageal cancer, 35 with gastric cancer, and 33 with colorectal cancer (N=95, **Table 1**). There were no statistically significant differences between the non-irAE group and the irAE group regarding the demographic/clinical information, such as gender, age, BMI, allergy history, tumor type, tumor stage, performance status, microsatellite status, number of prior lines of treatment, and immunotherapy drugs (**Table 1**). Forty-four patients reported immune-related adverse events with varying organ toxicity and severity (**Table 2**). Among them, the median time to first irAE occurrence was 27 days (min to max days: 1-212), and 35 (79.5%) patients developed irAEs within 12 weeks after receiving immunotherapy.

**Table 2:** Patient irAE information.

Organ toxicity (Total occurrences)	irAE	Occurrences	Grade 1	Grade 2	Grade ≥3
Skin toxicity (n=21)	Rash	11	9	1	1
	Pruritus	7	7	0	0
	Hair loss	2	2	0	0
	Hemangioma	1	0	1	0
Blood toxicity (n=14)	Leukopenia	5	5	0	0
	Neutropenia	2	1	0	1
	Anemia	6	4	2	0
	Thrombocytopenia	1	0	1	0
Hepatotoxicity (n=20)	ALT elevation	7	5	0	3
	AST elevation	7	5	1	2
	Bilirubin elevation	4	2	2	0
Gastrointestinal toxicity (n=2)	Diarrhea	2	2	0	0
Endocrine toxicity (n=12)	Hypothyroidism	5	3	2	0
	Hyperthyroidism	5	4	1	0
	Adrenal insufficiency	1	0	0	1
	Hypercalcemia	1	0	0	1
Myotoxicity (n=4)	Myositis	4	2	0	2
Pulmonary toxicity (n=1)	Interstitial pneumonia	1	1	0	0
Renal toxicity (n=8)	Proteinuria	8	5	2	1
Cardiac toxicity (n=1)	Premature atrial beats	1	1	0	0

### Gut microbiome composition was correlated with the occurrence of immune-related adverse events

**Figure 2:**
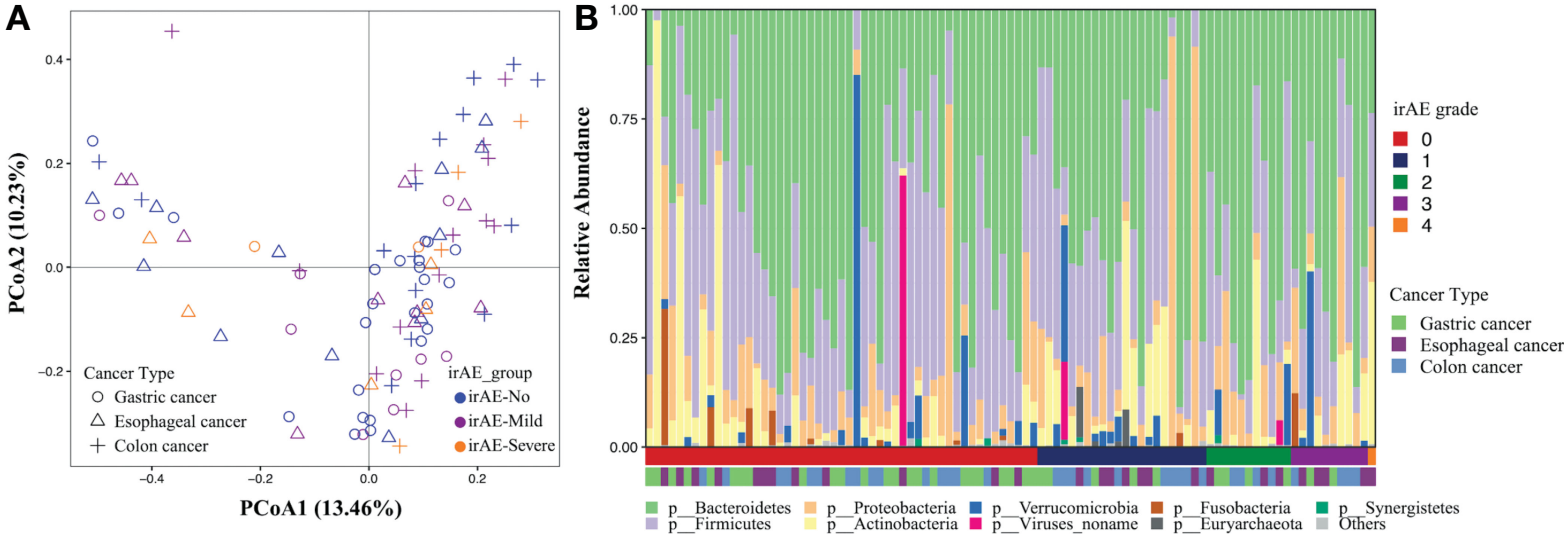
Gut microbial composition associated with immune-related adverse events. The analysis was performed on all patients. **(A)** Principal coordinates analysis of the microbial community based on the Bray-Curtis distance calculated at the species level. Each dot represents a stool sample with shape and color indicating cancer types and the severity of irAE. **(B)** Phylogenetic composition of common bacterial taxa at the phylum level, ordered by the irAE grade (the first horizontal bar underneath) and cancer types (the second horizontal bar underneath).

We first evaluated the gut microbiome of the baseline fecal samples in patients with and without irAE at the community level. No significant differences were observed in either alpha (both richness and evenness, data not shown) or beta diversity between the two groups, indicating there might be individual bacterial taxa related to irAE instead of the overall community shift (**Figure 2A**).

**Figure 3:**
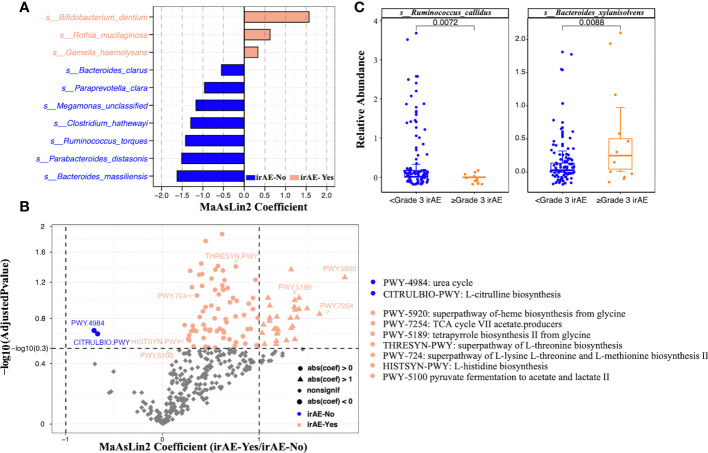
Significantly differential species and metabolic pathways between irAE and non-irAE groups. The analysis was performed on all patients. **(A)** Butterfly plot of differential bacterial species between patients with and without irAEs. The x axis shows the effect size represented by MaAsLin2 coefficient (positive number means enrichment in the irAE group; negative number means enrichment in the non-irAE group). **(B)** Volcano plot of microbial metabolic pathways. The x axis shows the effect size represented by MaAsLin2 coefficient (positive number means enrichment in the irAE group; negative number means enrichment in the non-irAE group). The dashed horizontal line shows adjusted P value of 0.3 and the two dashed vertical line showed coefficients of -1 and 1. **(C)** Boxplots showing the relative abundance of *Ruminococcus callidus* and *Bacteroides xylanisolvens* in the mild (<grade3) and severe (≥grade3) irAE groups. Wilcoxon rank sum test was applied, and p values were labeled for each comparison.

After PERMANOVA confirmed there was no significant correlation between patients’ demographic/clinical features with the gut microbiome, we applied differential analysis to identify the specific microbial taxa related to irAE (**Figure 2B**; **Supplementary Table 4**). Species such as *Clostridium hathewayi, Ruminococcus torques, Bacteroides massiliensis*, *Paraprevotella clara*, *Parabacteroides distasonis* and *Megamonas* were enriched in patients without irAEs (**Figure 3A**). Meanwhile, *Bifidobacterium dentium*, *Rothia mucilaginosa* and *Gemella haemolysans* were significantly higher in irAE patients (**Figure 3A**). Because metagenomics sequencing enables microbial functional level exploration, we thus compared MetaCyc metabolic pathways between patients with and without irAE. We also identified some metabolic pathways that were statistically different between the irAE group and the non-irAE group (**Figure 3B**). Among them, urea cycle (PWY-4984) and citrulline biosynthesis (CITRULBIO-PWY) were enriched in the non-irAE group, while glycine metabolism either in the super pathway of heme b biosynthesis from glycine (PWY-5920) or tetrapyrrole biosynthesis from glycine (PWY-5189), threonine and methionine biosynthesis (THRESYN-PWY, PWY-724), histidine biosynthesis (HISTSYN-PWY), pyruvate fermentation to acetate and lactate (PWY-5100) and TCA cycle VII acetate producers (PWY-7254) were significantly enriched in participants with irAEs. Notably, the above-mentioned *R. mucilaginosa*, *B. dentium* and *G. haemolysans* contributed to most of the metabolic pathways.

The occurrence of irAE was further divided into mild (grades 1-2) and severe (≥ grade 3), because mild symptoms can mostly be recovered with clinical intervention and severe irAEs (≥ grade 3) may related to serious clinical consequences and the patients have limited survival benefits due to ICI withdrawal. Analysis of the data indicated that *Ruminococcus callidus* and *Bacteroides xylanisolvens* further helped to distinguish the population with grade ≥3 irAEs (**Figure 3C**).

### irAE in different cancer types were associated with different gut microbiota profiles

**Figure 4:**
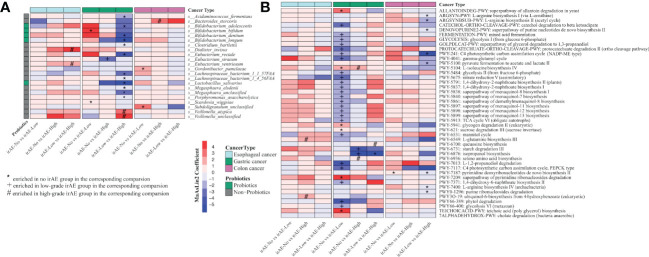
Significantly differential species and metabolic pathways in patients with different cancer types stratified by irAE severity. **(A)** Differential species between each comparison (irAE groups: irAE_No, irAE_Low and irAE_High) for esophageal cancer, colon cancer, and gastric cancer patients. Commonly considered probiotic species (green color) based on Probio https://bidd.group/probio/homepage.htm. **(B)** Differential metacyc pathway between each comparison (irAE groups: irAE_No, irAE_Low and irAE_High) for esophageal cancer, colon cancer and gastric cancer patients. MaAsLin2 was used to identify differential species and pathway between irAE groups. Each column indicates a comparison, and the labels (*,#,+) indicate the direction of enrichment. enrichment. Different colors of the rows indicate different cancer types.

We further explored the differences in gut microbiota in patients with different cancer types (**Supplementary Table 5**). In patients with esophageal cancer, regardless of whether the patients had immune-related adverse reactions, there were no significant differences in the alpha diversity or beta diversity of the gut microbiota at baseline (**Supplementary Figure 1**). *Ruminococcus torques* was enriched in the non-irAE group while *Dialister invisus* and *Eubacterium ventriosum* were enriched in the high irAE group compared to the low-grade irAE group (**Figure 4A**). At the pathway level, we found ubiquinol-6 biosynthesis (PWY3O-19) and glutamine biosynthesis (PWY-6549) were significantly enriched in the high irAE group (**Figure 4B**).

Among gastric cancer patients with irAEs, the gut microbiome showed reduced but not yet significant alpha diversity along with the irAE severity (**Supplementary Figure 2A**). Microbial community was significantly different among groups (low grade vs high grade: PERMANOVA p=0.046, **Supplementary Figure 2B**). Notably, multiple common probiotics species were enriched in the non-irAE group. At the family level, *Lactobacillaceae*, *Bifidobacteriaceae* and *Eubacteriaceae* were enriched in the non-irAE or low-grade irAE group (**Supplementary Figure 2C**). At the species level, we identified a few probiotic species enriched in no-irAE or low-irAE group, such as *Lactobacillus salivarius*, *Bifidobacterium longum*, *Bifidobacterium dentium*, *Bifidobacterium adolescentis*, *Bifidobacterium bifidum* (**Figure 4A**). In addition, several butyrate producers, such as *Eubacterium rectale*, *Megasphaera elsdenii* etc were also enriched in these groups (**Figure 4A**). At the pathway level, isopropanol biosynthesis (PWY-6876) and multiple pathways in menaquinol biosynthesis (PWY-5838, PWY-5840, PWY-5897, PWY-5898, PWY-5899) were enriched in no-irAE or low-irAE group (**Figure 4B**).

In patients with colon cancer, there were no significant differences in the alpha diversity and beta diversity of the gut microbiota in each group at baseline (**Supplementary Figure 3**). At the species level, *Bacteroides stercoris* were enriched in the high-grade irAE group (**Figure 4A**). At the pathway level, arginine biosynthesis (ARGSYNBSUB-PWY, ARGSYN-PWY, PWY-7400), and pyruvate fermentation to acetate and lactate pathway (PWY-5100) were significantly higher in low-grade irAE group (**Figure 4B**).

### Differences in gut microbiota profiles were associated with specific toxicity

**Figure 5:**
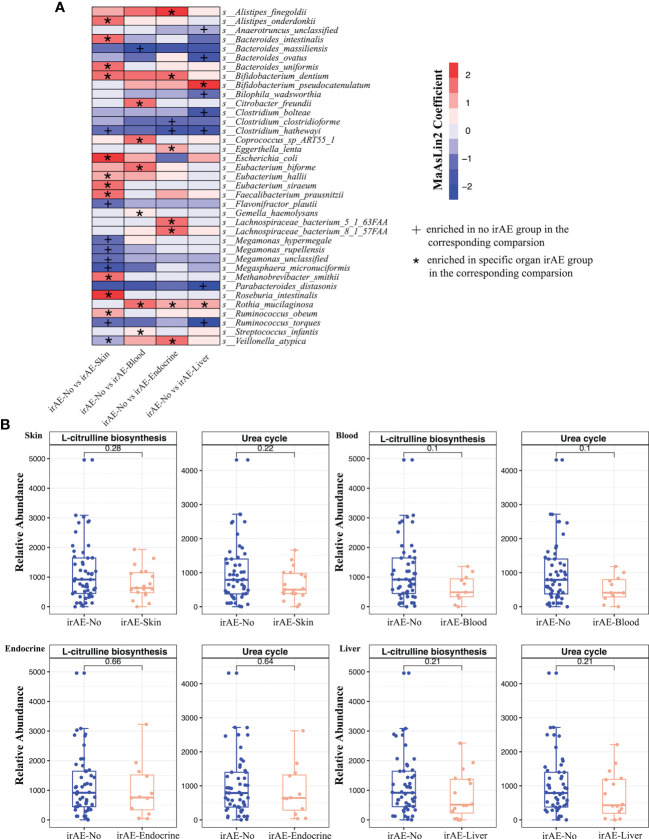
**(A)** Differential species between irAE and non-irAE patients for each comparison group. Each column indicates a comparison for the specific toxicity and the labels (*, +) indicate the direction of enrichment. **(B)** Boxplots showing the relative abundance of microbial metabolic pathways in irAE patients across different toxicities. Wilcoxon rank sum test was applied, and p values were labeled for each comparison.

Considering that irAEs in different organs may involve different mechanisms, we next compared the relationship between specific toxicity (skin, blood, endocrine, and liver) and the gut microbiota composition in different subgroups of irAE patients (**Supplementary Table 6**; **Supplementary Figure 4**–**7**). For skin irAEs, *Methanobrevibacter smithii*, *Bifidobacterium dentium*, *Roseburia intestinalis* and *Faecalibacterium prausnitzii* were enriched in the irAE group, while *Megasphaera micronuciformi*, *Clostridium hathewayi*, *Ruminococcus torques* and *Flavonifractor plautii* were enriched in the non-irAE group (**Figure 5A**). Out of total 111 metabolic pathways, glycerol degradation pathway (GOLPDLACT-PWY) and urea cycle (PWY-4984) were enriched in the non-irAE group with a major contribution from *F. plautii* (**Figure 5B**). For hematologic irAEs, *Bacteroides massiliensis* was enriched in the non-irAE group while notably, *Akkermansia* was enriched in gastric cancer patients with hematologic irAEs (**Figure 5A** **;** **Supplementary Figure 5C**). We also found urea cycle (PWY-4984) and citrulline biosynthesis (CITRULBIO-PWY) were significantly enriched in the non-irAE group (**Figure 5B** **;** **Supplementary Figure 8** **)**.

For endocrine irAEs, two *Clostridium* species (*Clostridium clostridioforme* and *Clostridium hathewayi*) were enriched in the non-irAE group, while *Alistipes finegoldii*, *Veillonella atypica* and *Lachnospiraceae* bacterium were increased in the irAE group (**Figure 5A**). Meanwhile, significant increases of the enterobactin biosynthesis pathway (ENTBACSYN-PWY) and O-antigen biosynthesis pathway (PWY-7328) were observed in the non-irAE group (**Supplementary Figure 8**). Lastly, for liver irAEs, *Ruminococcus torques*, *Parabacteroides distasonis* and *Clostridium hathewayi* were enriched in the non-irAE group compared to the enrichment of *Bifidobacterium pseudocatenulatum* in irAE patients (**Figure 5**). The significantly enriched pathways in the non-irAE group included urea cycle (PWY-4984) and citrulline biosynthesis (CITRULBIO-PWY), the same as in hematologic irAEs (**Figure 5B** **;** **Supplementary Figure 8**).

## Discussion

In this prospective study, we comprehensively analyzed the association between the gut microbiome and irAEs in 95 gastrointestinal cancer patients treated with immune checkpoint inhibitors and identified bacterial species and metabolic function pathways that were closely associated with irAEs.

Among the bacterial biomarkers related to irAEs, we found that *Ruminococcus callidus* was enriched in people without severe irAEs. A recent study reported that increased abundance of *R. callidus* was associated with favorable response to anti-PD-1 therapy and improved survival in hepatobiliary tumors (Mao et al., 2021). *Ruminococcus* was also reported to be associated with immune-related enteropathy and other immune diseases such as allergy, eczema, asthma, and other diseases (Chaput et al., 2017; Gopalakrishnan et al., 2018; Park et al., 2018), indicating its potential role in immune-modulation. We also found higher quantities of *Lactobacillus* and *Bifidobacterium* in gastric cancer patients with non- or low-grade irAEs. Many species of *Lactobacillus* and *Bifidobacterium* are considered to be probiotics that provide health benefits to the host. In addition to the prophylactic effects, their involvement in immunotherapy is starting to be noticed in recent years. Three studies on non-small cell lung cancer (NSCLC) patients showed an increase of *Lactobacillus* and/or *Bifidobacterium* in non or low-grade irAE cases (Hakozaki et al., 2020; Cascone et al., 2021; Chau et al., 2021).In a study of 70 Japanese NSCLC patients treated with PD-1/PD-L1, Hakozaki T et al. found that baseline samples from patients with no or grade 1 irAEs were enriched in *Lactobacillus* (Hakozaki et al., 2020). In a prospective cohort study from the United States, the researchers compared baseline fecal samples from 33 advanced non-small cell lung cancer patients with 32 healthy controls and analyzed the gut microbiome using 16S rRNA sequencing. *Bifidobacterium* was associated with lower severity of irAEs (Chau et al., 2021). In the NEOSTAR trial, Cascone et al. also observed an association between decreased toxicity to nivolumab and *Bifidobacterium* (Cascone et al., 2021). Similarly, in one preclinical study, Wang et al. found the abundance of *Lactobacillus* was significantly reduced in mice with immune-associated colitis (Satoh et al., 2020). Regarding the potential mechanisms, different species confer beneficial effects through various ways. Tan et al. demonstrated that *Lactobacillus rhamnosus* alleviated immune-related enteritis by regulating Treg cells in the mouse model (Tan et al., 2020). A study on *Lactobacillus reuteri* showed that it can prevent immune enteritis by reducing the number of group 3 innate lymphocytes (ILC3s) (Satoh et al., 2020). Preclinical studies also suggested *Bifidobacterium* supplementation could alleviate colitis in mice receiving anti-CLTA-4 (Wang et al., 2018), and this was potentially mediated by gut microbiome optimization, thereby enhancing the expression of IL-10Ra and IL-10 of intestinal Treg cells and ultimately alleviating immune-related intestinal damages (Sun et al., 2020).

Quite a few studies focusing on irAEs in melanoma patients showed decreased microbial diversity and potential bacterial biomarkers such as *Lachnospiraceae, Streptococcaceae* as well as several *Bacteroides* species (*B. dorei, B. vulgatus, B. intestinalis*) (Dubin et al., 2016; Chaput et al., 2017; Andrews et al., 2021; Liu et al., 2021; Usyk et al., 2021; McCulloch et al., 2022). Andrews et al. further demonstrated in preclinical models that the higher abundance of *B. intestinalis* promoted irAE toxicity through the upregulation of IL-1beta (Andrews et al., 2021). In the current study, however, we did not observe any bacterial diversity associated with irAEs and identified rather different bacterial biomarkers. This could potentially be due to variations in sequencing/analysis methods, cohort characteristics, treatment regimens, etc. because even within melanoma studies, we found multiple inconsistencies between published studies. Moreover, we fully acknowledge the gut microbiome profile is unique to each cancer type, as we have shown that even within gastrointestinal cancers, the gut microbiome of colorectal, gastric, and esophageal cancer patients are significantly different. Thus, gut microbial signatures related to each cancer type or even each specific cohort might be different, and this should be noted in current clinical practices.

Besides the above-mentioned microbial biomarkers, their encoded functions are also of great interest because the underlying mode of action on how gut microbiome impact irAEs might overlap among different cancer types despite the heterogeneous microbial biomarkers. In the current study, we showed pathways involved in the urea cycle, including citrulline and arginine biosynthesis, were associated with irAEs. Previous research showed urea cycle dysregulation and arginine metabolism play an important role in immunotherapy (Kim et al., 2018). Oral supplementation of arginine could significantly increase the efficacy of cyclophosphamide combined with anti-PD1 antibody in a mouse model (Satoh et al., 2020). Specifically, by engineering a probiotic strain to convert ammonia to arginine, Canale et al. showed that the enhanced anti-tumor effect was mediated by arginine and dependent on T cells (Canale et al., 2021). Although the role of urea cycle in irAEs has not been reported, our data indicated its potential connections and warrants further examination. In addition, a recent study demonstrated that a gut ecosystem enriched with beneficial microbial functions and a richer butyrate production pathway was significantly associated with a reduced incidence of irAEs in melanoma patients (Maung et al., 2020). Rectal or oral butyrate-based therapy has shown promising results in intestinal inflammatory diseases (Vernia et al., 2000; Hallert et al., 2003; Di Sabatino et al., 2005). In our study, although we did not find significant enrichment of butyrate-producing pathways in non- or low-grade irAE, a decent number of butyrate producers were identified. For example, significant enrichment of *Eubacterium rectale* and *Megasphaera elsdenii* was observed in non-/low irAE gastric cancer patients.

In this study, we investigated the correlation between gut microbiota and irAEs in 95 gastrointestinal cancer patients. With the help of metagenomic sequencing, we identified bacterial species and metabolic pathways that might be associated with the occurrence of irAEs in gastric, esophageal, and colon cancers and across multiple organs. We believe this work provides a foundation for future mechanism exploration and clinical applications. Despite these exciting findings, we also acknowledge that there are several limitations of this study, including the lack of external validation cohorts as well as experimental validation of the potential mechanisms we hypothesized.

## Data availability statement

The datasets presented in this study can be found in online repositories. The names of the repository/repositories and accession number(s) can be found below: NCBI sra BioProject: PRJNA910239.

## Ethics statement

The studies involving human participants were reviewed and approved by Ethics Committee of Beijing Cancer Hospital. The patients/participants provided their written informed consent to participate in this study.

## Author contributions

YZ: Resources, data curation, investigation, methodology, writing–original draft. SC: Resources, data curation, investigation, and manuscript writing. HZ: data curation, data analysis, manuscript writing. ZH, TX, BZ: data curation, investigation, reviewed and revised the manuscript. DD: data curation, data analysis. YL implemented the bioinformatics workflow and technical support. XY, YK, YT: research design, analysis supervise, reviewed, and revised the manuscript. LS, ZP: Conceptualization, supervision, funding acquisition. All authors contributed to the article and approved the submitted version.
