# Supplementary figures and images for: Correlation of the gut microbiome and immune-related adverse events in gastrointestinal cancer patients treated with immune checkpoint inhibitors
Source: Front Cell Infect Microbiol. 2023 Mar 3;13:1099063. doi: 10.3389/fcimb.2023.1099063 (PMC10084768; doi:10.3389/fcimb.2023.1099063)

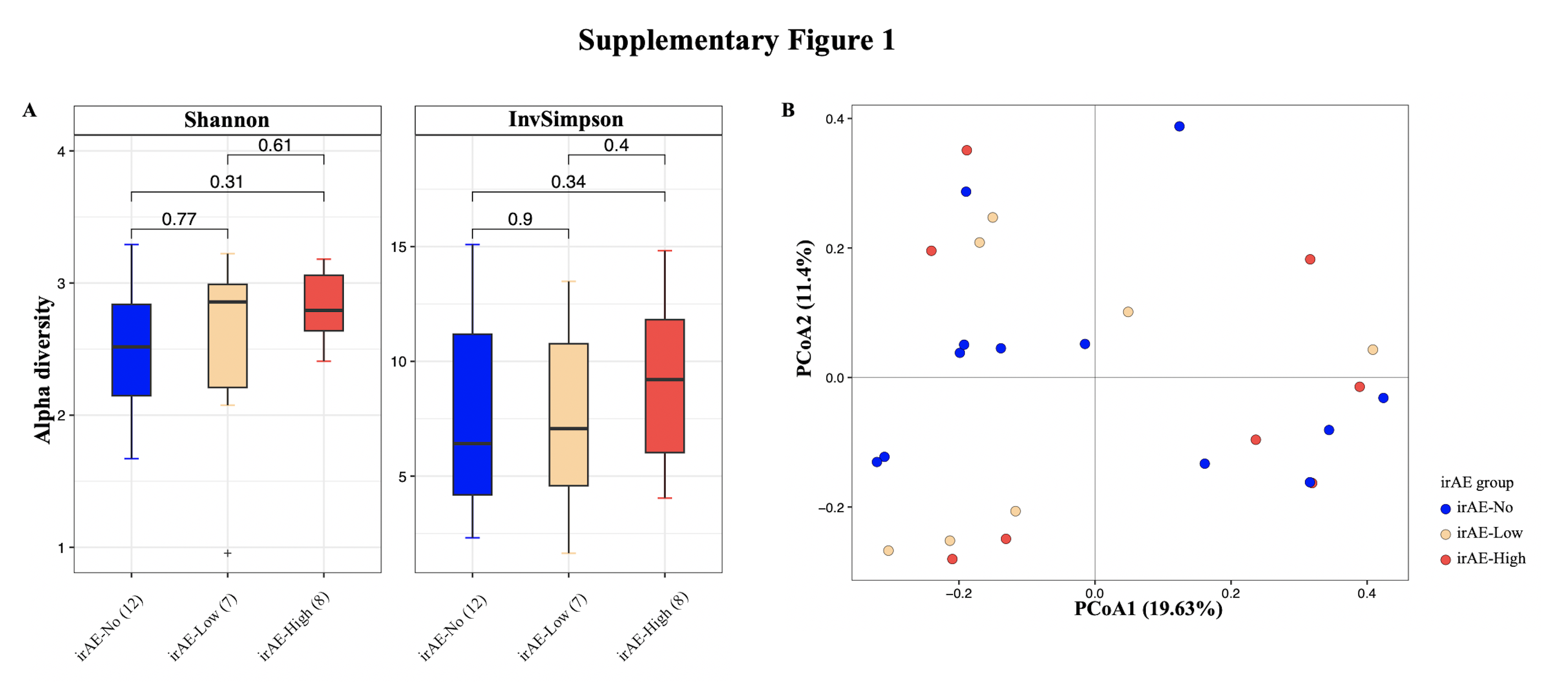

Supplement: Supplementary file 7 [file Image_1.jpeg]

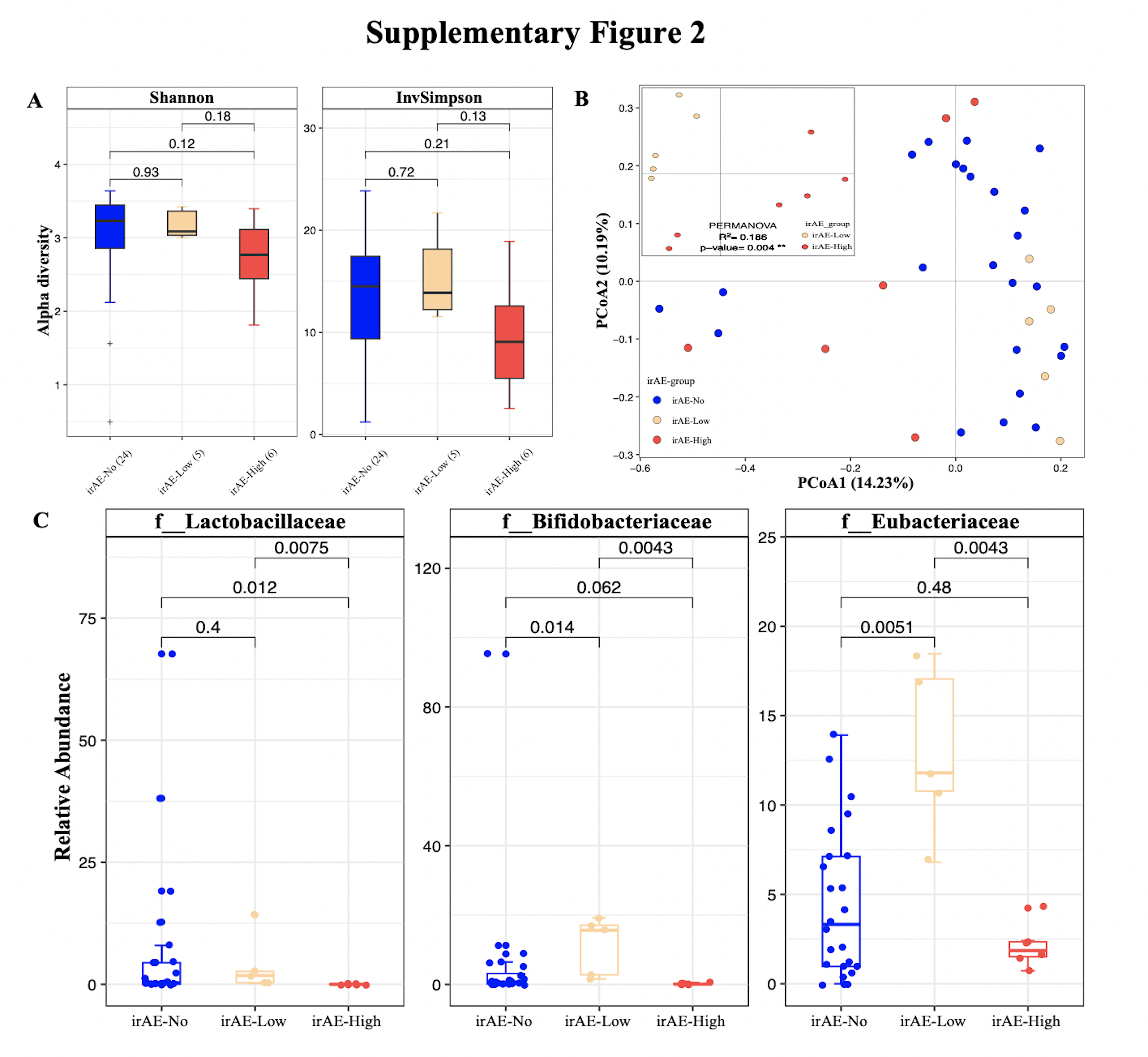

Supplement: Supplementary file 8 [file Image_2.jpeg]

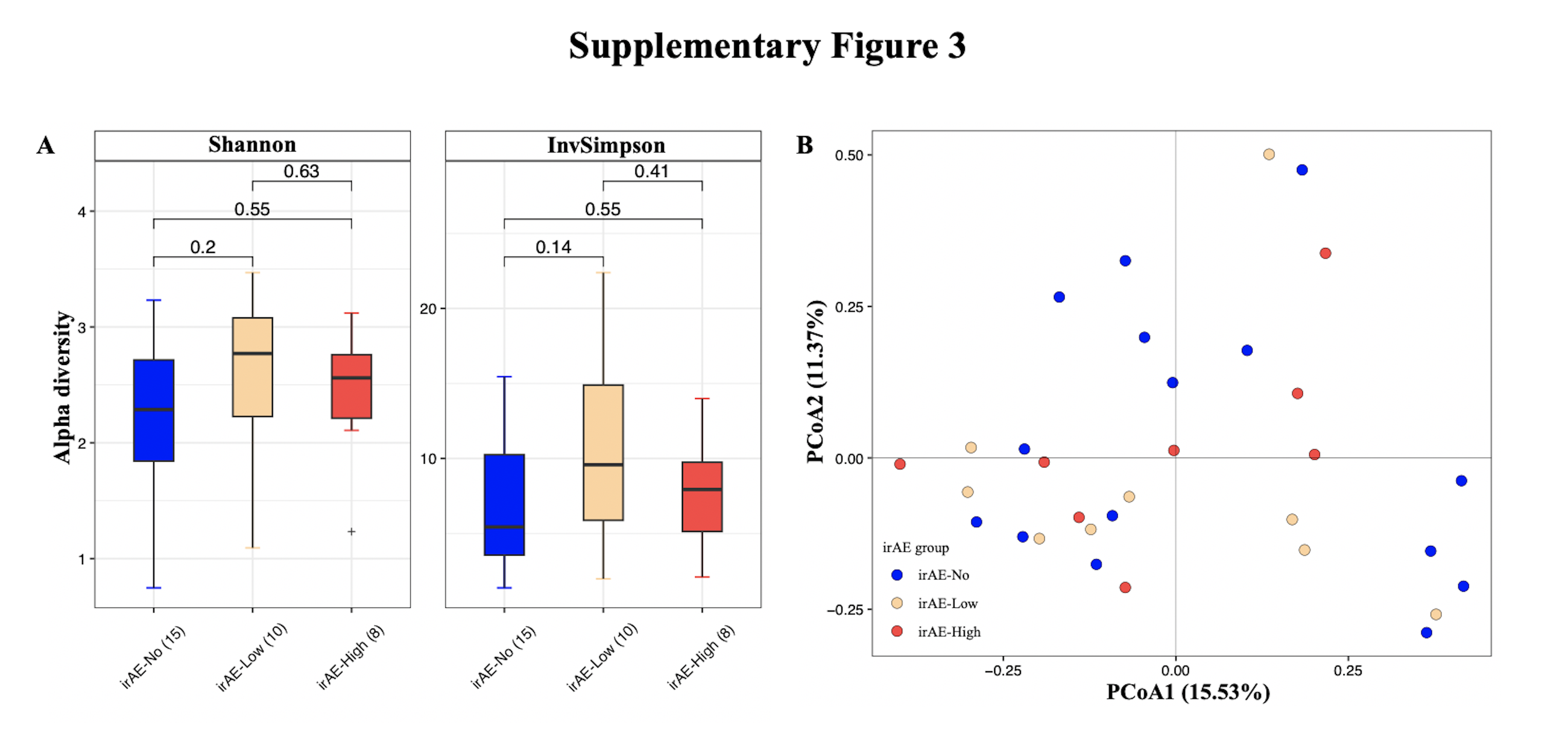

Supplement: Supplementary file 9 [file Image_3.jpeg]

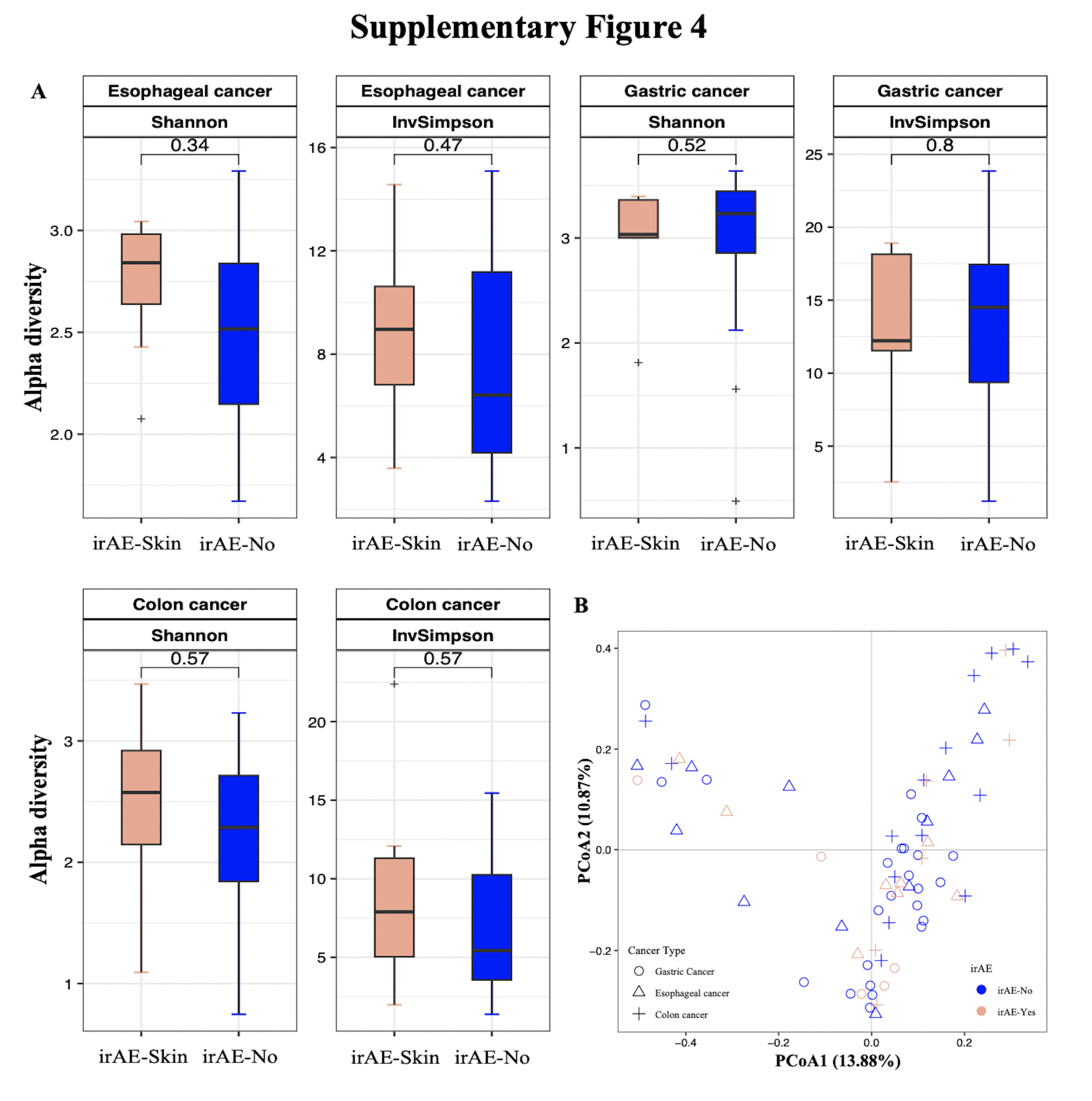

Supplement: Supplementary file 10 [file Image_4.jpeg]

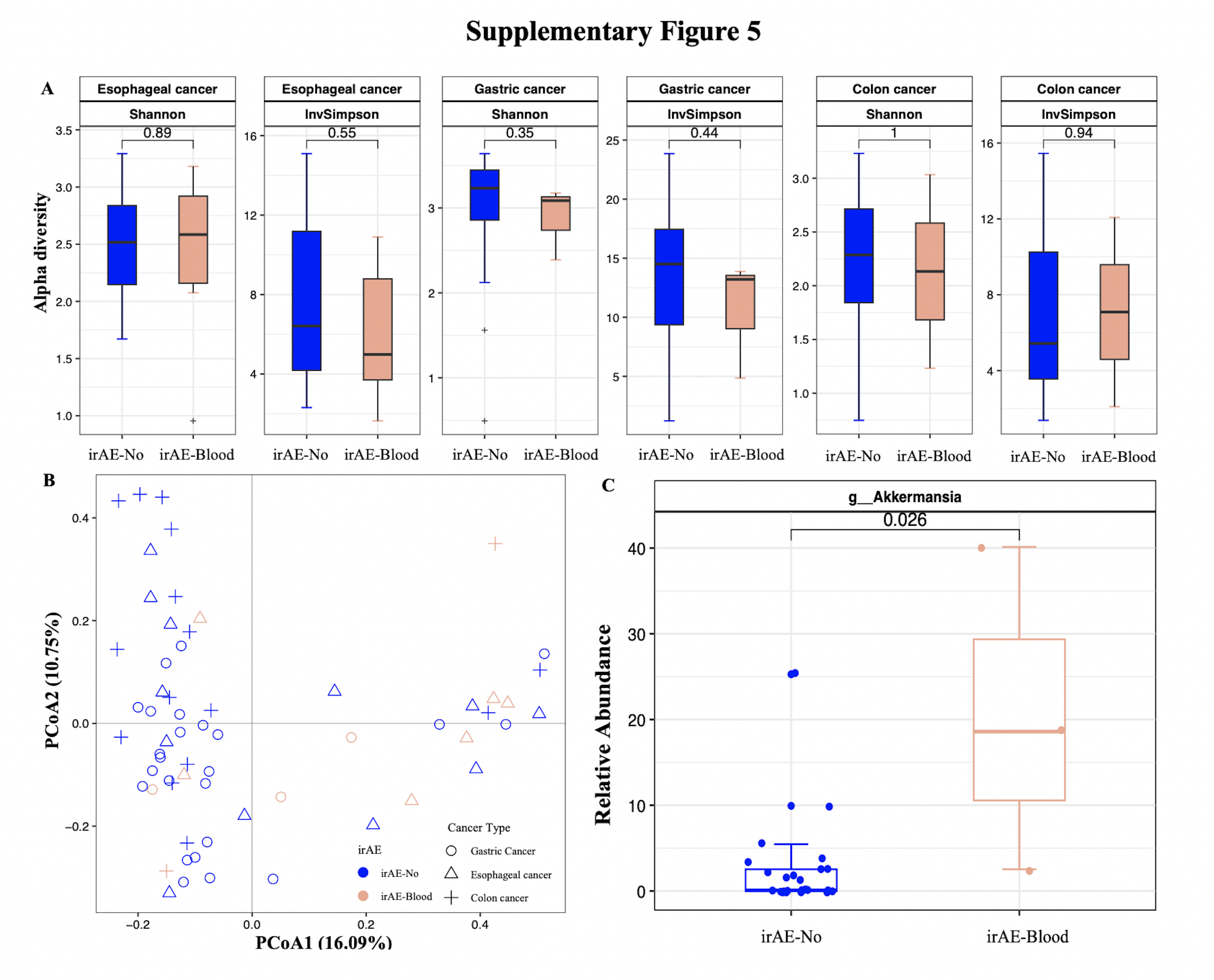

Supplement: Supplementary file 11 [file Image_5.jpeg]

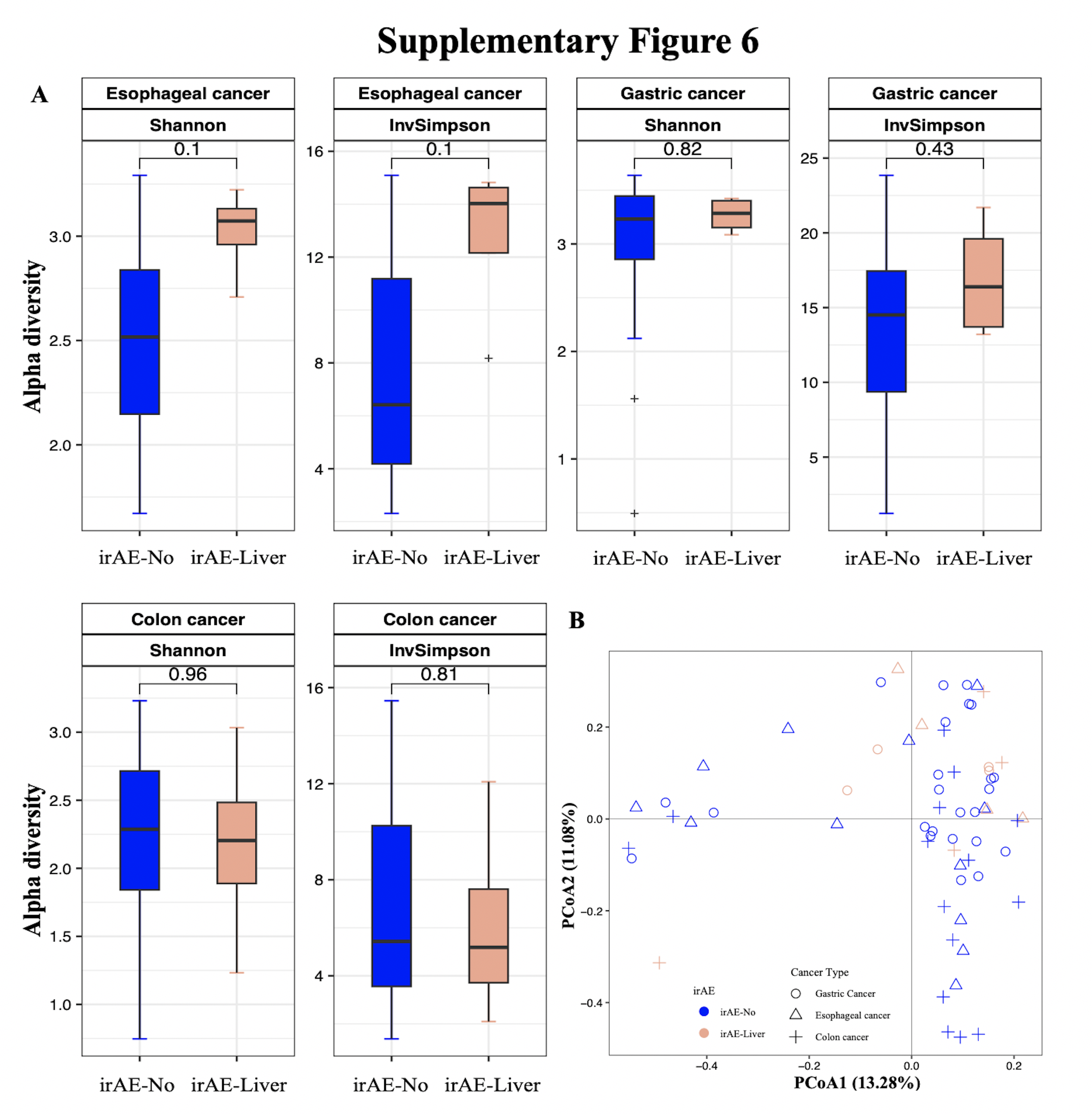

Supplement: Supplementary file 12 [file Image_6.jpeg]

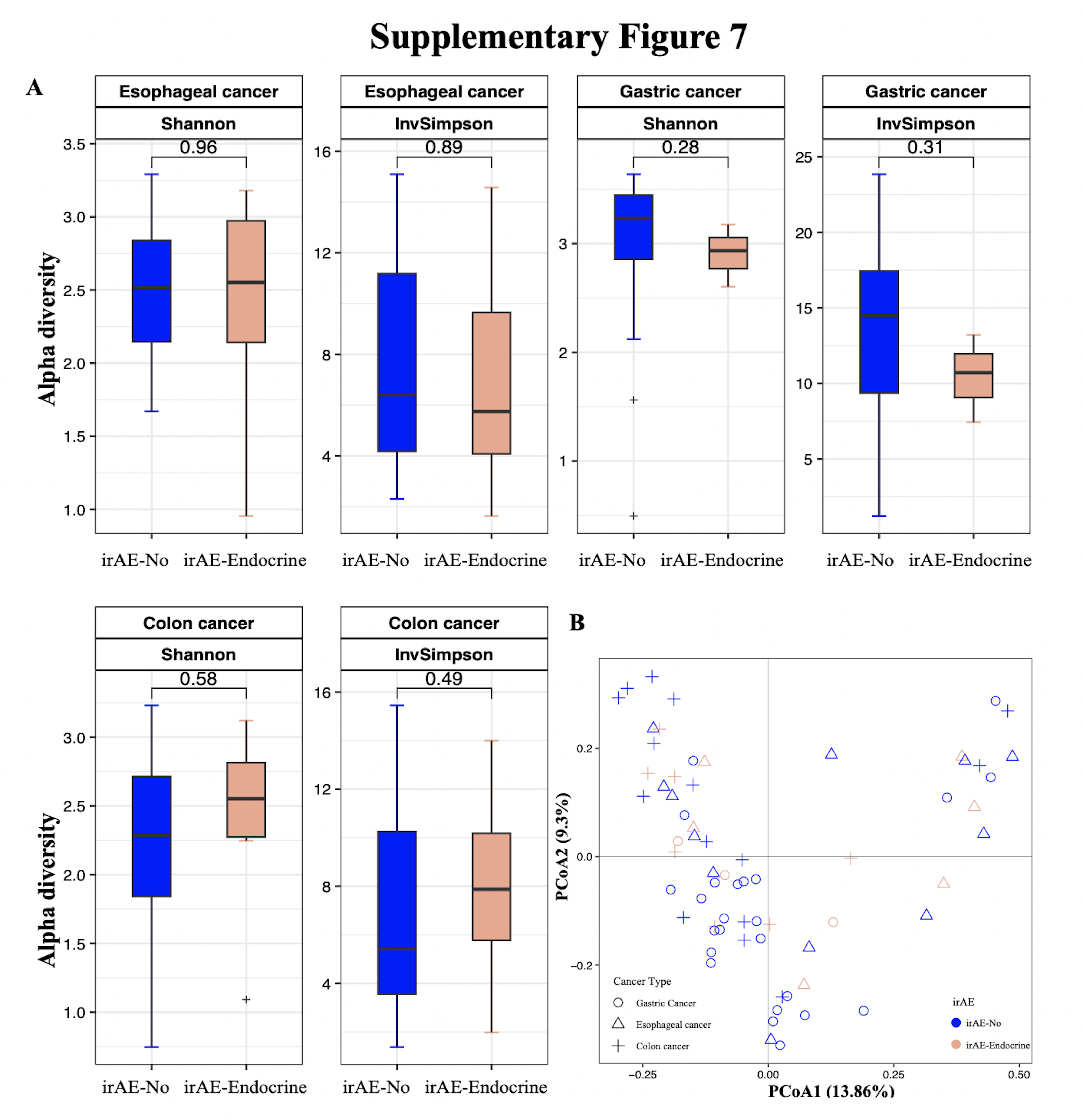

Supplement: Supplementary file 13 [file Image_7.jpeg]

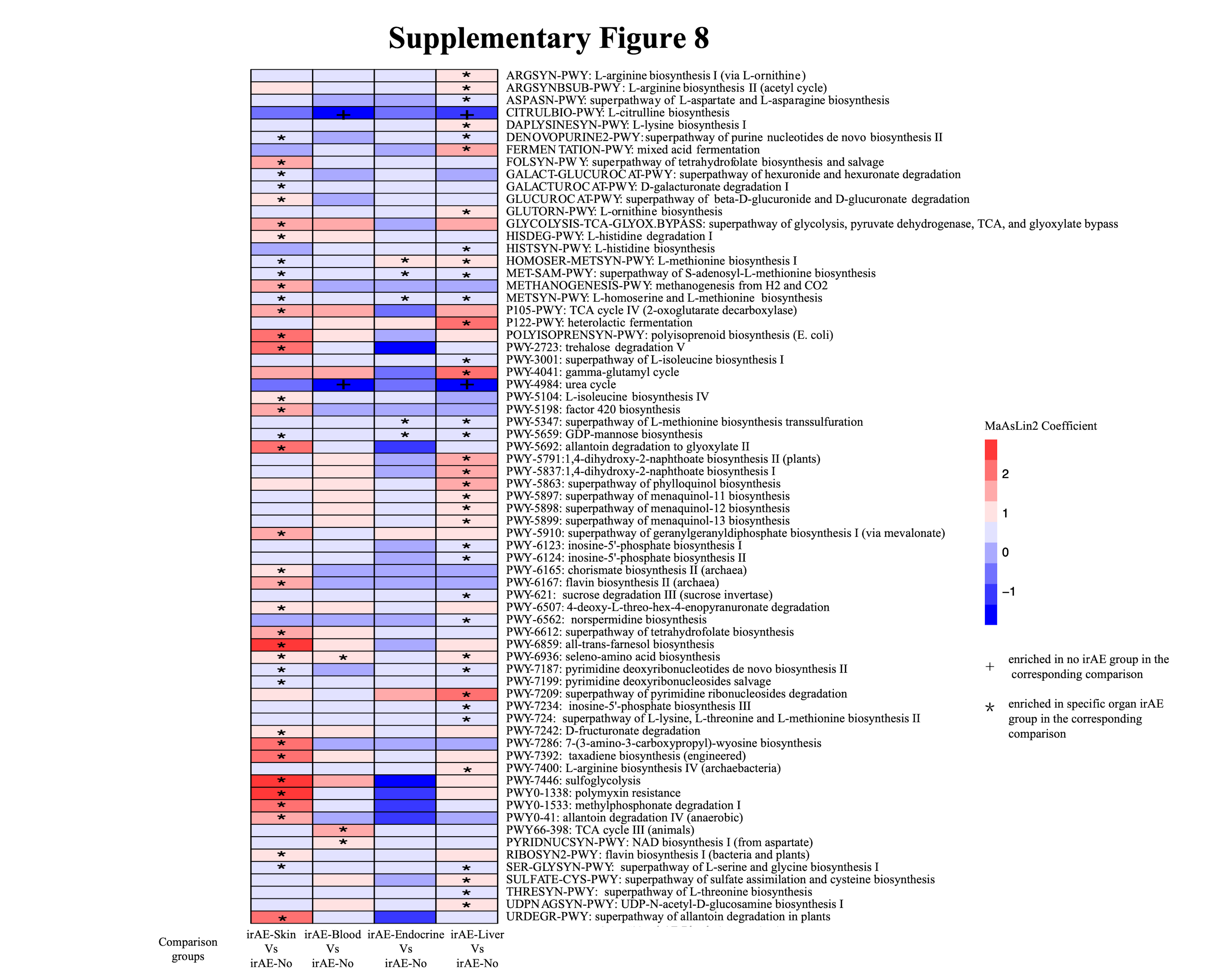

Supplement: Supplementary file 14 [file Image_8.jpeg]
